# Robust Synchronization of Delayed Chaotic FitzHugh-Nagumo Neurons under External Electrical Stimulation

**DOI:** 10.1155/2012/230980

**Published:** 2012-11-01

**Authors:** Muhammad Rehan, Keum-Shik Hong

**Affiliations:** ^1^Department of Electrical Engineering, Pakistan Institute of Engineering and Applied Sciences (PIEAS), P.O. Box 45650, Islamabad, Pakistan; ^2^Department of Cogno-Mechatronics Engineering and School of Mechanical Engineering, Pusan National University, 30 Jangjeon-Dong, Geumjeong-Gu, Busan 609-735, Republic of Korea

## Abstract

Synchronization of chaotic neurons under external electrical stimulation (EES) is studied in order to understand information processing in the brain and to improve the methodologies employed in the treatment of cognitive diseases. This paper investigates the dynamics of uncertain coupled chaotic delayed FitzHugh-Nagumo (FHN) neurons under EES for incorporated parametric variations. A global nonlinear control law for synchronization of delayed neurons with known parameters is developed. Based on local and global Lipschitz conditions, knowledge of the bounds on the neuronal states, the Lyapunov-Krasovskii functional, and the *L*_2_ gain reduction, a less conservative local robust nonlinear control law is formulated to address the problem of robust asymptotic synchronization of delayed FHN neurons under parametric uncertainties. The proposed local control law guarantees both robust stability and robust performance and provides the *L*_2_ bound for uncertainty rejection in the synchronization error dynamics. Separate conditions for single-input and multiple-input control schemes for synchronization of a wide class of FHN systems are provided. The results of the proposed techniques are verified through numerical simulations.

## 1. Introduction 

The neuron is the fundamental unit of the functioning brain [1]. Its dynamical investigation, for the purpose of measuring brain activity and understanding how the neural system transmits electrochemical signals to the muscles, is one of the most significant challenges facing brain researchers [2–8]. Neural-system malfunctions can cause potentially fatal motor-function-impairment diseases such as Parkinson's, Huntington's, and epilepsy [9–11]. Synchronization of chaotic neurons with gap junctions under external electrical stimulation (EES), one of the most fundamental research issues, has been relentlessly studied in order to improve therapy-based treatments of neurodegenerative disorders [12, 13]. In this respect, the FitzHugh-Nagumo (FHN) model, which has been applied in other fields (e.g., chemical reaction kinetics [14]) as well, is one of the most pertinent neural models utilized in synchronization studies [15–18]. 

 Given the FHN model's wide applicability, many of its significant and complex dynamical aspects, including chaos, bifurcation, synchronization, control, noise effects and filtering, coupling, and medium effects, not to mention disturbance rejection, have already been reviewed extensively in the literature [12–20]. Researchers have applied nonlinear, adaptive, fuzzy, neural-network-, and observer-based, as well as robust control methodologies to the synchronization of FHN neurons under EES [12–14, 16, 21–24]. However, these traditional conservative synchronization controller design techniques [12–14, 16, 21–24], which in mathematical models ignore the time-delay arising due to the separation between coupled neurons, cannot synchronize distant FHN neurons. The dynamics of coupled chaotic delayed FHN neurons with gap junctions under EES recently have been investigated [17, 18], which can be accounted for synchronization studies. 

In the present work, we examined, preparatory to a brief numerical simulation study, the dynamics of coupled delayed FHN neurons in consideration of the model parametric variations. This parametric-variation-based model, with its separate visualization of each uncertain component, offers better insight into dynamical uncertainty in actual neurons; as such, it is a superior means of control law formulation for neuronal synchronization. Control law derivation for synchronization of delayed FHN neurons separated by gap junctions under EES has remained extremely rare to this date. In the interests of filling this research gap, we propose a global synchronization control strategy for identical neurons with known parameters. To that end, we also propose a novel local robust control law that guarantees asymptotic convergence of synchronization errors to zero under time-delays and parametric uncertainties. This regional control methodology, which is based on local and global Lipschitz constraints on nonlinear and uncertain components of neuronal dynamics, knowledge of state bounds, and the Lyapunov-Krasovskii (LK) functional, is less conservative in its performance within the desired locality. Additionally, we developed conditions under which robust control law performance bounds, which distinguish our work and enable the choice of a suitable robust controller, can be determined. The proposed computationally simple control strategy, with its easy design procedure, ensures both robust stability and robust performance in neuronal synchronization. Details on the robust single-input and multiple-input control strategies are presented herein to facilitate their application to a wide class of FHN systems. Finally, the proposed control schemes are successfully validated by numerical simulations. The main contributions of this paper are as follows.

- The dynamics of coupled delayed FHN neurons under parametric uncertainties are studied in order to provide better insight into uncertain coupled neurons.
- Global synchronization control of delayed FHN neurons, with guaranteed convergence of synchronization errors to zero, is achieved.
- A less conservative nonlinear control law for local robust synchronization of delayed FHN neurons under parametric uncertainties is developed that ensures asymptotic convergence of synchronization errors to zero.
- A robust performance assessment tool, in terms of the robustness bound, is provided for evaluation of the performance of a local controller.
- Single-input and multiple-input synchronization control laws that select specific and different objective functions for robust performance in their respective case, are derived in order to broaden the scope of the proposed schemes.

This paper is organized as follows.  Section 2 provides a brief overview of two identical coupled chaotic FHN neurons with gap junctions, delays, and parametric uncertainties.  Section 3 proposes a nonlinear control law for global synchronization of neurons with delays.  Section 4 addresses the issue of local robust synchronization of delayed uncertain FHN neurons.  Section 5 provides and discusses the simulation results for the proposed schemes.  Section 6 draws conclusions. 

Standard notations are used in this paper. The *L*_2_ norm of a vector *z* is defined as ||*z*||_2_ = (∫_0_^*∞*^||*z*||^2^ *dt*)^1/2^, where ||*z*|| denotes the Euclidian norm of *z*. A positive definite symmetric matrix *X* is denoted as *X* > 0. For *x*_*i*_ with the *i*th diagonal entry and *i* = 1,2,…, *n*, diag⁡(*x*_1_, *x*_2_,…, *x*_*n*_) represents a diagonal matrix. 

## 2. Model Description 

 Consider two identical uncertain coupled chaotic FHN neurons with time-delays under EES:

(1) dx1dt=x1(x1−1)(1−(r+Δr)x1)−y1−(g+Δg)(x1−x2(t−τ))+s1(t),dy1dt=(b+Δb)x1−(v+Δv)y1,dx2dt=x2(x2−1)(1−(r+Δr)x2)−y2−(g+Δg)(x2−x1(t−τ))+s2(t),dy2dt=(b+Δb)x2−(v+Δv)y2,

where *x* and *y* represent the states of a neuron in terms of activation potential and recovery voltage, respectively; (*x*_1_, *y*_1_) and (*x*_2_, *y*_2_) represent the states of the master and slave FHN neurons, respectively; *g* describes the strength of the gap junctions between neurons; the parameter *τ* > 0 indicates the time-delay due to separation between neurons. The master and the slave neurons are under external electrical stimulation with currents *s*_1_(*t*) = (*a*_1_/*ω*_1_)cos⁡*ω*_1_ *t* and *s*_2_(*t*) = (*a*_2_/*ω*_2_)cos⁡*ω*_2_ *t*, respectively. Here, *ω*_1_ = 2*πf*_1_ and *ω*_2_ = 2*πf*_2_ are dimensionless angular frequencies, *a*_1_/*ω*_1_ and *a*_1_/*ω*_1_ are stimulation amplitudes, and *t* denotes time. 

In biological models, we often know only the estimated or nominal values of parameters, not the exact or true values. Thus, in synchronizing identical neurons, the exact values of the model parameters, unlike the cases in scenarios examined in previous studies [12, 13, 21, 22], are unknown. In contrast to the literature [12–18, 21, 22], the terms Δ*g*, Δ*r*, Δ*b*, and Δ*v*, representing the parametric variations in *g*, *r*, *b*, and *v*, respectively, are added to the FHN neurons (1). Another reality of biological models is that the two neurons cannot be at all identical. Certainly, synchronization of identical coupled chaotic systems [25, 26], such as delayed FHN neurons under parametric uncertainties, has remained as a complex, challenging, and nontrivial problem. This problem can be resolved by utilization of adaptive control that is computationally complex [27, 28], and indeed especially complex when adaptation laws are required for a number of parametric variations. In order to resolve this problem, we now address the issue of robust nonlinear controller design. The model parameters selected are

(2) r=10,  g=0.1,  f1=f2=0.138,  b=1,a1=a2=0.1,  τ=20,  v=0.1

along with the parametric uncertainties

(3) $$\begin{array}{c} \Delta g=0.1,\;\Delta r=1,\;\Delta b=-0.1,\;\Delta v=-0.01. \end{array}$$

**Figure 1:**
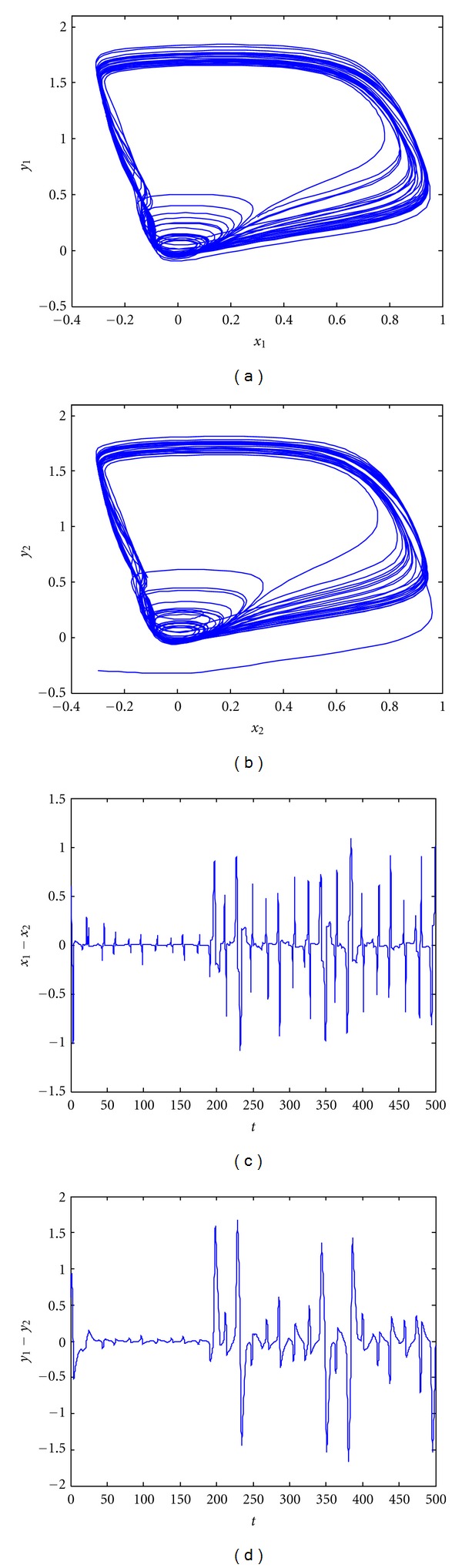
Chaotic behavior of nonsynchronous coupled delayed FHN neurons with parametric variations under EES. (a) Phase portrait of *x*_1_ and *y*_1_; (b) phase portrait of *x*_2_ and *y*_2_; (c) synchronization error *e*_1_ = *x*_1_ − *x*_2_ versus *t*; (d) synchronization error *e*_2_ = *y*_1_ − *y*_2_ versus *t*.

Figure 1 shows phase portraits and synchronization error plots for FHN neurons. These chaotic neurons are not synchronous, as the synchronization errors in Figures 1(a) and 1(b) do not converge to zero. We develop a control strategy utilizing two control inputs, *u*_1_ and *u*_2_, for synchronization of the slave neuron with the master neuron. The FHN model with control inputs, then, is given by

(4) dx1dt=x1(x1−1)(1−(r+Δr)x1)−y1−(g+Δg)(x1−x2(t−τ))+s1(t),dy1dt=(b+Δb)x1−(v+Δv)y1,dx2dt=x2(x2−1)(1−(r+Δr)x2)−y2−(g+Δg)(x2−x1(t−τ))+s2(t)+u1,dy2dt=(b+Δb)x2−(v+Δv)y2+u2.

Now, the design of control inputs *u*_1_ and *u*_2_ for synchronization of the FHN neurons will be addressed.

## 3. Global Nonlinear Control 

 The traditional control techniques have not clearly addressed synchronization of delayed FHN neurons, not even in the absence of uncertainty. Indeed, delayed neuronal models recently have been analyzed [17, 18], and their synchronization remains an open problem. We therefore develop a global nonlinear control strategy to address this issue. The proposed nonlinear control law for the synchronization of both FHN neurons is given by

(5) u1=C0(x1−x2)+C1(x1(t−τ)−x2(t−τ))−((1+r)x22−rx23)+((1+r)x12−rx13)+s1(t)−s2(t),u2=C2(y1−y2),

where *C*_0_, *C*_1_, and *C*_2_ are the controller gains. The term *C*_0_(*x*_1_ − *x*_2_) in the control law is required for convergence for the activation potential of the slave neuron to the corresponding state of the master neuron. The delayed term *C*_1_(*x*_1_(*t* − *τ*) − *x*_2_(*t* − *τ*)) provides an extra degree of freedom for convergence of the activation potential. Identical behavior of recovery variables of the two neurons can be achieved by application of *C*_2_(*y*_1_ − *y*_2_). The nonlinear components −((1 + *r*)*x*_2_^2^ − *rx*_2_^3^) and ((1 + *r*)*x*_1_^2^ − *rx*_1_^3^) are used to cancel the effect of known nonlinear parts in the synchronization error dynamics, which is required to simplify the controller design (see, e.g, [23, 24] and references therein). To overcome the effect of difference in stimulation signals for the master and the slave FHN neurons, the term *s*_1_(*t*) − *s*_2_(*t*) is used in the control law (5).

**Remark 1**

The proposed control law is computationally simpler than the so-called traditional synchronization techniques [12, 13, 21–23], despite the fact that it deals with FHN neuron dynamics that are more complex owing to delays and parametric uncertainties (discussed below). Moreover, to further reduce control workload and memory utilization, the parameters *C*_1_ and *C*_2_ can be selected as zero. These parameters are included in the control law to make it more general and, thus, applicable to other complex chaotic systems as well. Selection of *C*_2_ = 0 makes the controller input *u*_2_ = 0, which is desired for synchronization of FHN neurons. The techniques presented in this study, then, are useful for designing either a single-input or a multiple-input controller according to the given requirement.

To address the synchronization of neurons in the absence of uncertainties, we make the following assumption.

**Assumption 2**

The parametric uncertainties Δ*g*, Δ*r*, Δ*b*, and Δ*v* are zero.

The following theorem provides the sufficient condition for global synchronization of delayed chaotic FHN neurons.

**Theorem 3**

Suppose that the FHN neurons (4) satisfying Assumption 2, the nonlinear control law given by (5), synchronizes the coupled neurons asymptotically with proper selection of parameters *C*_0_, *C*_1_, and *C*_2_, if the matrix inequalities

(6) $$\begin{array}{c} P>0,\;\;Q>0,\;\;\Omega =\left[\begin{array}{cc} A^{T}P+PA+Q & PA_{1}\\ {}* & -Q \end{array}\right]<0 \end{array}$$

are satisfied, where

(7) A=[−(1+C0+g)−1b−(v+C2)],A1=[−(C1+g)000].

**Proof**

Using the control law (5) in (4) under Assumption 2, the overall closed-loop system becomes

(8) dx1dt=x1(x1−1)(1−rx1)−y1−g(x1−x2(t−τ))+s1(t),dy1dt=bx1−vy1,dx2dt=−rx13+(1+r)x12−x2−y2−g(x2−x1(t−τ))+s1(t)+C0(x1−x2)+C1(x1(t−τ)−x2(t−τ)),dy2dt=bx2−vy2+C2(y1−y2).

Defining the synchronization errors as

(9) $$\begin{array}{c} e_{1}=x_{1}-x_{2},\;\;e_{2}=y_{1}-y_{2}, \end{array}$$

the error dynamics are

(10) $$\begin{array}{c} \frac{de_{1}}{dt}=-\left(1+C_{0}+g\right)e_{1}-e_{2}-\left(C_{1}+g\right)e_{1}\left(t-\tau \right),\\ \frac{de_{2}}{dt}=be_{1}-\left(v+C_{2}\right)e_{2}. \end{array}$$

The error dynamics can be written as

(11) $$\begin{array}{c} \frac{de}{dt}=Ae+A_{1}e\left(t-\tau \right), \end{array}$$

where *e* = [*e*_1_ *e*_2_]^*T*^, *e*(*t* − *τ*) = [*e*_1_(*t*−*τ*) *e*_2_(*t*−*τ*)]^*T*^, and matrices *A* and *A*_1_ are given by (7). Now consider the Lyapunov functional

(12) $$\begin{array}{c} V\left(e,t\right)=e^{T}Pe+\int_{t-\tau }^{t}e^{T}\left(\zeta \right)Qe\left(\zeta \right)d\zeta , \end{array}$$

such that *P* > 0, *Q* > 0. The derivative of (12) along (11) is

(13) V˙(e,t)=eT(ATP+PA+Q)e+eT(t−τ)A1TPe+eTPA1e(t−τ)−eT(t−τ)Qe(t−τ).

For stability, $\dot{V}(e,t)<0$, that is, *Ω* < 0, which completes the proof of Theorem 3.

**Remark 4**

Traditional studies on control and synchronization of FHN models for a variety of applications ignored the factor of time-delays, which can produce unrealistic outcomes. In the present study, the consideration of time-delays due to separation between FHN systems such as neurons makes the synchronization problem more realistic than the classical schemes [12, 13, 21–23].

**Remark 5**

The proposed nonlinear control strategy utilizing matrix inequalities, derived by employing the LK functional, guarantees global asymptotic synchronization of delayed FHN neurons. These inequalities can be resolved, for a selection of control parameters, by using linear matrix inequality (LMI)-based tools. This makes the computation of matrices *P* and *Q* easier and hence helpful for selection of control parameters. It is also possible to incorporate additional LMI constraints for better performance, for instance to fix the upper bound on the synchronization error convergence rate.

## 4. Local Nonlinear Control

In the previous section, all parameters of FHN neurons were assumed to be known, with zero variations. In this section, we derive a sufficient condition for the synchronization of both FHN neurons under time-delays and parametric variations. Additionally, we provide sufficient conditions for the robust performance of single-input and multiple-input controllers. It is not always necessary to synchronize two identical oscillators globally. If the regional bounds on the states of the oscillators are known, a local controller can be a better choice. In reality, local controllers are less conservative, due to easy management of performance, robustness, and computation reduction for a given specific region (rather than emphasizing the whole space) [14, 29, 30]. Before delving into design methodology, we will review some basic definitions from the literature [31, 32].

**Definition 6**

The *L*_2_ gain of a system from signal *d* to *e* is said to be less than a positive scalar *γ* if ||*e*||_2_ < *γ*||*d*||_2_ + *β*, where *β* is a small positive constant [31].

**Definition 7**

A function *f*(*x*) is said to be Lipschitz if it satisfies the Lipschitz condition

(14) $$\begin{array}{c} ||f\left(x\right)-f\left(\overset{-}{x}\right)||\leq ||L\left(x-\overset{-}{x}\right)||, \end{array}$$

where $x,\overset{-}{x},L\in R$. Moreover, the Lipschitz nonlinearities also satisfy

(15) $$\begin{array}{c} ||\frac{\partial f}{\partial x}\left(x\right)||\leq ||L||, \end{array}$$

which is an inequality useful in determining *L*, by application of numerical algorithms.

**Definition 8**

A function *f*(*x*) is said to be locally Lipschitz for *x* ∈ *R* if it satisfies the conditions (14)-(15) locally for a bounded region $\left\{x,\overset{-}{x}\in [x_{\min},x_{\max}]:x_{\min},x_{\max}\in R\right\}$, where *x*_min⁡_ and *x*_max⁡_ are the minimum and maximum limits on *x* (or $\overset{-}{x}$), respectively.

From Definition 8, it can be seen that

(16) ||−x13+x12+x23−x22||≤||La(x1−x2)||,       ∀x1,x2∈[xmin⁡,xmax⁡],

where *L*_*a*_ is the Lipschitz constant for the local region, which can be selected by solving (15). To address the issue of the synchronization of neurons under parametric uncertainties, we make the following assumption.

**Assumption 9**

The parametric uncertainties are bounded by ||Δ*g*|| ≤ *g*_*m*_, ||Δ*b*|| ≤ *b*_*m*_, ||Δ*r*|| ≤ *r*_*m*_, and ||Δ*v*|| ≤ *v*_*m*_.

It is clear from (16) that the nonlinearity present in the FHN model satisfies the local Lipschitz condition. It has been reported previously [14, 29, 32] that a local controller can be designed for locally Lipschitz nonlinear systems if the bounds on the states of the system are known. Thanks to case studies, it is in fact well known that the states of a real neuron are always bounded in terms of the limits on activation potential and recovery voltage. This fact can be observed also in the simulation results shown in Figure 1 (see [12, 13, 17, 18, 21–24] as well). Therefore, by incorporating the knowledge of the minimum and maximum values of the states of neurons, the idea of bounds *g*_*m*_, *b*_*m*_, *r*_*m*_, and *v*_*m*_, and by noting the fact that the nonlinear part of the dynamics is locally Lipschitz, a regional robust controller can be designed for synchronization of FHN neurons.

**Theorem 10**

Suppose that the FHN neurons (4) satisfy Assumption 9 with states bounded by a region *x*_1_, *x*_2_ ∈ [*x*_min⁡_, *x*_max⁡_]. The nonlinear control law (5) synchronizes the coupled neurons asymptotically with proper selection of parameters *C*_0_, *C*_1_, and *C*_2_, if the following matrix inequalities are verified:

(17) Ps>0,  Qs>0,Γs=[ATP+PA+Q+F1PA1PPP∗−Q+F2000∗∗−I00∗∗∗−I0∗∗∗∗−I]<0,

where

(18) $$\begin{array}{c} F_{1}=\left[\begin{array}{cc} r_{m}^{2}L_{a}^{2}+g_{m}^{2}+b_{m}^{2} & 0\\ 0 & v_{m}^{2} \end{array}\right],\;\;F_{2}=\left[\begin{array}{cc} g_{m}^{2} & 0\\ 0 & 0 \end{array}\right], \end{array}$$

and matrices *A* and *A*_1_ are given by (7).

**Proof**

Incorporating the control law (5) into (4), the overall closed-loop system becomes

(19) dx1dt=x1(x1−1)(1−(r+Δr)x1)−y1−(g+Δg)(x1−x2(t−τ))+s1(t),dy1dt=(b+Δb)x1−(v+Δv)y1,dx2dt=−rx13+(1+r)x12−x2−y2−(g+Δg)×(x2−x1(t−τ))+s1(t)+C0(x1−x2)+C1(x1(t−τ)−x2(t−τ))−Δrx23+Δrx22,dy2dt=(b+Δb)x2−(v+Δv)y2+C2(y1−y2).

Using the same procedure as discussed in the previous section, the following error dynamics model is obtained:

(20) $$\begin{array}{c} \frac{de}{dt}=Ae+A_{1}e\left(t-\tau \right)+\Psi +\Theta +\Phi , \end{array}$$

where

(21) Ψ=ψ(x1,y1)−ψ(x2,y2),Θ=θ(x1)−θ(x2),Φ=ϕ(x1(t−τ))−ϕ(x2(t−τ)),ψ(x1,y1)=[−Δr(x13−x12)−Δgx1−Δvy1],ψ(x2,y2)=[−Δr(x23−x22)−Δgx2−Δvy2],θ(x1)=[0Δbx1],  θ(x2)=[0Δbx2],ϕ(x1(t−τ))=[−Δgx1(t−τ)0],ϕ(x2(t−τ))=[−Δgx2(t−τ)0].

On the basis of Assumption 9 and inequality (16), we have

(22) ||Δr(−x13+x12+x23−x22)||≤||rmLa(x1−x2)||,          ∀x1,x2∈[xmin⁡,xmax⁡],||Δg(−x1+x2)||≤||gm(x1−x2)||.

Combining the local and global Lipschitz constraints of (22), we have

(23) ||Δr(−x13+x12+x23−x22)+Δg(−x1+x2)|| ≤(rm2La2+gm2)||(x1−x2)||, ∀x1,x2∈[xmin⁡,xmax⁡].

As we know from the global Lipschitz condition,

(24) ||Δb(x1−x2)||≤||bm(x1−x2)||,

(25) ||Δv(−y1+y2)||≤||vm(y1−y2)||,

(26) ||Δg(−x1(t−τ)+x2(t−τ))||  ≤||gm(x1(t−τ)−x2(t−τ))||.

Inequalities (23)–(25) imply

(27) $$\begin{array}{c} \Psi^{T}\Psi +\Theta^{T}\Theta \leq e^{T}F_{1}e, \end{array}$$

and (26) implies

(28) $$\begin{array}{c} \Phi^{T}\Phi \leq e^{T}\left(t-\tau \right)F_{2}e\left(t-\tau \right). \end{array}$$

Constructing the LK functional

(29) $$\begin{array}{c} E\left(e,t\right)=e^{T}P_{s}e+\int_{t-\tau }^{t}e^{T}\left(\zeta \right)Q_{s}e\left(\zeta \right)d\zeta , \end{array}$$

with *P*_*s*_ > 0 and *Q*_*s*_ > 0.

Taking the derivative of (29) along (20)

(30) E˙(e,t)=eT(ATPs+PsA+Qs)e+eT(t−τ)×A1TPse+eTPsA1e(t−τ)−eT(t−τ)×Qse(t−τ)+ΨTPse+eTPsΨ+ΘTPse+eTPsΘ+ΦTPse+eTPsΦ,

and, using inequalities (27)-(28), we obtain

(31) E˙(e,t)≤eT(ATPs+PsA+Qs)e+eT(t−τ)A1TPse+eTPsA1e(t−τ)−eT(t−τ)Qse(t−τ)+ΨTPse+eTPsΨ+ΘTPse+eTPsΘ+ΦTPse+eTPsΦ−ΨTΨ−ΘTΘ−ΦTΦ+eTF1e+eT(t−τ)F2e(t−τ).

This further implies that

(32) $$\begin{array}{c} \dot{E}\left(e,t\right)\leq \xi^{T}\Gamma_{s}\xi , \end{array}$$

where

(33) ξT=[eTeT(t−τ)ΨTΘTΦT]T.

For stability, $\dot{E}(e,t)<0$, and hence Γ_*s*_ < 0, which completes the proof of Theorem 10.

**Remark 11**

It is notable that Theorem 10 also guarantees global asymptotic synchronization in the absence of parametric uncertainties. By ignoring Φ, Ψ, and Θ in (31), corresponding to Δ*g* = 0, Δ*r* = 0, Δ*b* = 0, and Δ*v* = 0, one can obtain the matrix inequalities in (6).

Theorem 10 provides a sufficient condition for the local robust asymptotic synchronization of uncertain delayed FHN neurons, ensuring zero synchronization errors in the steady state. The other pertinent issue is robust performance in terms of the *L*_2_ gain reduction from the uncertain nonlinearities Ψ, Θ, and Φ to the error *e*. By selecting a controller with a smaller size of the error *e* with respect to the uncertainties Ψ, Θ, and Φ, the required robust performance can be achieved. For this purpose, again consider system (20), but in an alternative form given by

(34) dedt=Ae+A1e(t−τ)+[III]d, with  d=[ΨTΘTΦT]T,

where *I* represents the identity matrix of appropriate dimensions. Although the asymptotic convergence of error *e* to zero under parametric uncertainties can be ensured by Theorem 10, the performance of the synchronization control can be improved for robustness with the help of additional constraints addressing the minimization of the effects of uncertainties in *d* at error *e* (see also [33–35]). To that end, we provide a sufficient condition for robust asymptotic synchronization of FHN neurons with robustness bound *γ* in terms of the *L*_2_ gain from the uncertain nonlinearities to the error.

**Theorem 12**

Consider FHN neurons (4) satisfying Assumption 9 with states bounded by a region *x*_1_, *x*_2_ ∈ [*x*_min⁡_, *x*_max⁡_]. Suppose the optimization problem

(35) $$\begin{array}{c} \min\gamma , \end{array}$$

such that

(36) $$\begin{array}{c} P_{s}>0,\;Q_{s}>0,\;\Gamma_{s}<0, \end{array}$$

(37) Pr>0, Qr>0, γ>0,Γr=[ATPr+PrA+QrPrA1PrPrPrI∗−Qr0000∗∗−γI000∗∗∗−γI00∗∗∗∗−γI0∗∗∗∗∗−γI]<0.

Then, the control law (5), with proper selection of parameters *C*_0_, *C*_1_, and *C*_2_ ensures the following.

- asymptotic synchronization of neurons with zero steady state synchronization error;
- the *L*_2_ gain from the nonlinear uncertainties *d* to the error *e* less than *γ*.

**Proof**

Consider the objective function

(38) $$\begin{array}{c} \min\gamma \end{array}$$

such that

(39) E˙(e,t)<0,J(e,t)=E˙r(e,t)+(1/γ)eTe−γdTd<0,

where

(40) Er(e,t)=eTPre+∫t−τteT(ζ)Qre(ζ)dζ>0,   with  Pr>0, Qr>0,

and *E*(*e*, *t*) > 0, as already defined in (29). It has already been shown, in Theorem 10, that $\dot{E}(e,t)<0$ leads to matrix inequalities in (36), ensuring the robust asymptotic synchronization of neurons. Hence *e*(*t*) → 0 as *t* → *∞*, which completes the proof of statement (i) in Theorem 12. Now, integrating *J* from *t* = 0 to *t* → *∞*, and multiplying by *γ*, we obtain

(41) $$\begin{array}{c} {||e||}_{2}^{2}<\gamma^{2}{||d||}_{2}^{2}+\gamma \left(E_{r}\left(e,0\right)-E_{r}\left(e,\infty \right)\right). \end{array}$$

From (40), we have *E*_*r*_(*e*, 0) > 0, because *P*_*r*_ > 0, *Q*_*r*_ > 0, and *E*_*r*_(*e*, *∞*) = 0 (given that *e*(*t*) → 0 as *t* → *∞*). Accordingly, (41) shows that the *L*_2_ gain from *d* to *e* is less than *γ*. Taking the derivative of (40) along (34) and incorporating it into (39), we obtain

(42) J(e,t)=eT(ATPr+PrA+Qr)e+eT(t−τ)A1TPre+eTPrA1e(t−τ)−eT(t−τ)Qre(t−τ)+ΨTPre+eTPrΨ+ΘTPre+eTPrΘ+ΦTPre,+eTPrΦ+(1/γ)eTe−γ(ΨTΨ+ΘTΘ+ΦTΦ)

which further can be written as

(43) $$\begin{array}{c} J\left(e,t\right)=\zeta^{T}\Pi \;\zeta <0, \end{array}$$

where

(44) Π=[ATPr+PrA+Qr+(1/γ)IPrA1PrPrPr∗−Qr000∗∗−γI00∗∗∗−γI0∗∗∗∗−γI]<0.

Using the Schur complement, inequality (44) can be written as Γ_*r*_ < 0, which completes the proof of statement (ii) in Theorem 12.

**Remark 13**

The sufficient condition for synchronization of FHN neurons, provided by Theorem 12, ensures ||*e*||_2_^2^ < *γ* ^2^||*d*||_2_^2^ + *γ*(*E*_*r*_(*e*, 0)) as *E*_*r*_(*e*, *∞*) = 0. Note that *E*_*r*_(*e*, 0) is dependent on initial condition *e*(0). Therefore, the effects of the uncertain nonlinear terms contained in *d* and the initial-condition-dependent term *E*_*r*_(*e*, 0) are minimized by minimizing *γ*. Hence, the proposed condition, by managing a single parameter *γ*, ensures robustness against both the uncertainties and the initial condition.

**Remark 14**

Recently, global synchronization of FHN models with unknown parameters and in the absence of time-delays, by application of a nonlinear robust adaptive control methodology, was presented [23]. Such a control scheme is computationally complex due to the utilization of adaptation laws. In the present work, synchronization of delayed FHN models under parametric uncertainties is addressed by designing a noncomplex locally robust controller.

In neuronal synchronization, only measurements of the activation potentials and control input *u*_1_ are available, though the robust synchronization control addressed by Theorem 10 is general for selection of a control law with a single control input *u*_1_ (by taking *C*_2_ = 0) or multiple control inputs *u*_1_ and *u*_2_. However, Theorem 12, providing robust synchronization along with robust performance, is better for the two-control-input case in which the second control input requires measurement of recovery potentials. For a single control input, it is better to ensure the minimization of the *L*_2_ gain from *d* to *e*_1_ than from *d* to *e*, for three reasons. First, the most relevant uncertain neuronal state is the activation potential due to uncertainty in *r* and *g*. Because *e*_1_ = *x*_1_ − *x*_2_, the above-noted criterion is helpful when dealing with variations in activation potentials. The second reason is that we have no control input to handle the uncertainties Δ*b* and Δ*v* (because, in the case of neurons, *u*_2_ = 0 with a single-input controller). And third, we can still ensure the robustness of *e*_2_ by minimizing the *L*_2_ gain from *d* to *e*_1_, because the dynamical equation ${\dot{e}}_{2}=(b+\Delta b)e_{1}-(v+\Delta v)e_{2}$ also contains *e*_1_. This indicates that the robustness of *e*_1_ will, somehow, ensure the robustness of *e*_2_. Although obtaining matrix inequalities for this performance criterion is a straightforward extension of Theorem 12, this important issue remains unaddressed in the literature; we prefer therefore to apply the results for this case to the following Theorem.

**Theorem 15**

Given FHN neurons (4) satisfying Assumption 9 with states bounded by a region *x*_1_, *x*_2_ ∈ [*x*_min⁡_, *x*_max⁡_], suppose the optimization problem

(45) $$\begin{array}{c} \min\gamma , \end{array}$$

such that

(46) Ps>0, Qs>0, Γs<0,Pr>0, Qr>0, γ1>0,[ATPr+PrA+QrPrA1PrPrPrdiag⁡(1,0)∗−Qr0000∗∗−γ1I000∗∗∗−γ1I00∗∗∗∗−γ1I0∗∗∗∗∗−γ1I]<0.

Then, the control law (5), with proper selection of parameters *C*_0_ and *C*_1_ along with *C*_2_ = 0, ensures

- asymptotic synchronization of neurons with zero steady state synchronization error;
- the *L*_2_ gain from the nonlinear uncertainties *d* to the error *e*_1_ less than *γ*_1_.

**Proof**

The proof is similar to that of Theorem 12.

**Remark 16**

The proposed techniques provide both “robust stability” (in terms of asymptotic convergence of synchronization errors to zero) and “robust performance” (in terms of uncertainty rejection) as addressed by Theorems 10–15. In contrast to the traditional schemes, the robust performance of the proposed synchronization schemes, addressed in Theorems 12–15, is an extra feature of the proposed controller (in addition to asymptotic stabilization).

**Remark 17**

The present work, in contrast to traditional synchronization techniques for FHN neurons, is novel in many respects. Our techniques consider time-delay between interlinked neurons. The easy LMI-based means of control parameter selection is another key characteristic, as is the exceptional idea of a less conservative local robust nonlinear controller for local synchronization of uncertain delayed FHN neurons. Our work furthers the design of both single- and multiple-input controllers by addressing their robust performances.

**Remark 18**

The synchronization techniques proposed by Theorems 3–15 for FHN models can be used for a number of purposes. These techniques can be applied to estimate the control signal *u*_1_ responsible for synchronization of two uncertain delayed neurons, which can be helpful in future for measuring brain activity and for improving stimulation-therapy-based treatments for brain disorders. The proposed schemes for synchronization of two delayed neurons can be generalized to deal with a delayed neural network. Further, such methodologies can be used for biomimetic systems in order to develop artificial neural networks, which can be useful for humanoid robotic applications. Furthermore, synchronization studies, owing to the capability of the FHN model to represent complex processes such as the reaction-diffusion system, can be applied to control (or synchronize) (the chemical kinematics of) industrial plants under time-delays.

Theorem 12 facilitates the selection of a suitable multiple-input robust controller with guaranteed robust stability, ensured by constraints (36), as well as robust performance in terms of *L*_2_ gain *γ*, ensured by constraints (37).  Theorem 15 provides further results for the special and important case of a single-input robust controller. The traditional techniques, with the help of additional constraints, have not yet adequately addressed robust stability and robust performance simultaneously for synchronization of FHN neurons. In future, the ideas presented in this work will be extended to address the issue of robust synchronization of different uncertain delayed neurons.

## 5. Simulation Results

To confirm the validity of the proposed schemes, we choose multiple- and single-input controllers *K*_I_ and *K*_II_, respectively.

(47) KI={C0=10,C1=0,C2=10.

(48) KII={C0=17,C1=0,C2=0.

**Figure 2:**
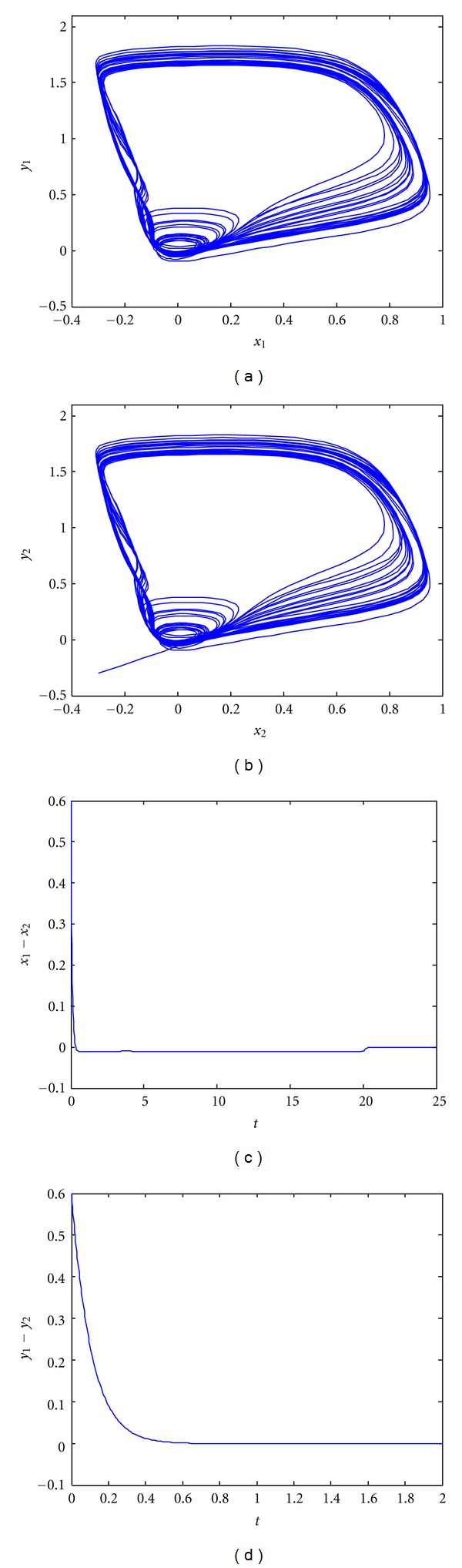
Synchronization of delayed coupled FHN neurons with parametric uncertainties using robust multiple-input controller *K*_I_. (a) Phase portrait of *x*_1_ and *y*_1_; (b) phase portrait of *x*_2_ and *y*_2_; (c) synchronization error *e*_1_ = *x*_1_ − *x*_2_ versus *t*; (d) synchronization error *e*_2_ = *y*_1_ − *y*_2_ versus *t*.

Note that these controllers are global in the absence of parametric uncertainties and local otherwise, due to the feasibility of both Theorems 3 and 10. Moreover, both controllers *K*_I_ and *K*_II_ have no memory feedback, because *C*_1_ = 0. By solving Theorem 12, the *L*_2_ gain *γ* = 0.141 for *K*_I_ is obtained. Local synchronization of the FHN models is considered for the region [*x*_min⁡_, *x*_max⁡_] = [−0.5,1] with *L*_*a*_ = 2. Phase portraits and synchronization error plots for *K*_I_ are shown in Figure 2. Clearly, the FHN neurons are synchronized with zero steady-state synchronization errors, demonstrating suitable robust performance under the parametric uncertainties. 

**Figure 3:**
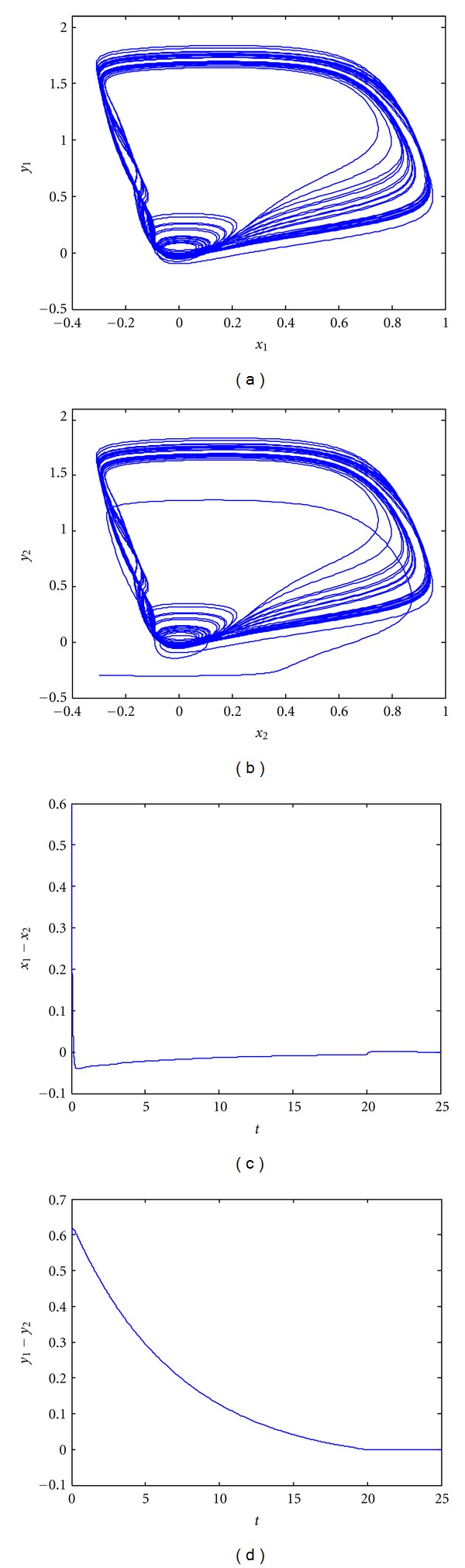
Synchronization of delayed coupled FHN neurons with parametric uncertainties using robust single-input controller *K*_II_. (a) Phase portrait of *x*_1_ and *y*_1_; (b) phase portrait of *x*_2_ and *y*_2_; (c) synchronization error *e*_1_ = *x*_1_ − *x*_2_ versus *t*; (d) synchronization error *e*_2_ = *y*_1_ − *y*_2_ versus *t*.

The controller *K*_I_, though robust, requires two control inputs in addition to measurement of the recovery potentials. We then check the performance of controller *K*_II_ for the same region [*x*_min⁡_, *x*_max⁡_] = [−0.5,1] and *L*_*a*_ = 2. The *L*_2_ gain *γ*_1_ = 0.508 is obtained by solving Theorem 15.  Figure 3 provides phase portraits and synchronization error plots validating the results obtained by Theorem 15. The performance of controller *K*_II_ is not better than that of *K*_I_, though we use a higher value of gain *C*_0_ for *K*_II_. This is due to the lack of a second control input *u*_2_ in the single-input control case. Nonetheless, the results obtained using *K*_II_, which offers additional computational simplicity with no requirement for multiple control inputs or measurement of recovery potentials, are reasonable.

## 6. Conclusions

This paper provided a brief look at coupled delayed FHN neurons with various parametric variations under EES. A global nonlinear control law was developed for the asymptotic synchronization of delayed FHN neurons. By integrating the ideas of neuronal state bounds, local and global Lipschitz conditions for the nonlinear and uncertain components of the dynamics of delayed neurons, the LK functional, and *L*_2_ gain reduction, a less conservative regional robust synchronization control was developed that ensures both robust stability and robust performance. Computational simplicity, a simple design procedure, guaranteed zero steady-state synchronization error, a computed robustness bound and applicability to both multiple- and single-input controllers are additional distinguishing features of the proposed schemes. Simulations of the uncertain coupled chaotic delayed FHN neuronal synchronization validated the proposed methodology.
